# Unraveling the nature of sensing in electrostatic MEMS gas sensors

**DOI:** 10.1038/s41378-024-00688-3

**Published:** 2024-05-06

**Authors:** Yasser S. Shama, Sasan Rahmanian, Hamza Mouharrar, Rana Abdelrahman, Alaaeldin Elhady, Eihab M. Abdel-Rahman

**Affiliations:** 1 Systems Design Engineering, University of Waterloo, 200 University Ave W, Waterloo, N2L 3G1 ON Canada; 2 Mechanical Engineering, Benha Faculty of Engineering, Benha University, Benha, 13511 Egypt

**Keywords:** Physics, Engineering

## Abstract

This paper investigates the fundamental sensing mechanism of electrostatic MEMS gas sensors. It compares among the responsivities of a set of MEMS isopropanol sensors before and after functionalization, and in the presence and absence of electrostatic fields when operated in static and dynamic detection modes. In the static mode, we found that the sensors do not exhibit a measurable change in displacement due to added mass. On the other hand, bare sensors showed a clear change in displacement in response to isopropanol vapor. In the dynamic mode, functionalized sensors showed a measurable frequency shift due to the added mass of isopropanol vapor. In the presence of strong electrostatic fields, the measured frequency shift was found to be threefold larger than that in their absence in response to the same concentration of isopropanol vapor. The enhanced responsivity of dynamic detection allows the sensors to measure the vapor mass captured by the functional material, which is not the case for static detection. The detection of isopropanol by bare sensors in static mode shows that change in the medium permittivity is the primary sensing mechanism. The enhanced responsivity of dynamic mode sensors when operated in strong electrostatic fields shows that their sensing mechanism is a combination of a weaker added mass effect and a stronger permittivity effect. These findings show that electrostatic MEMS gas sensors are independent of the direction of the gravitational field and are, thus, robust to changes in alignment. It is erroneous to refer to them as ‘gravimetric’ sensors.

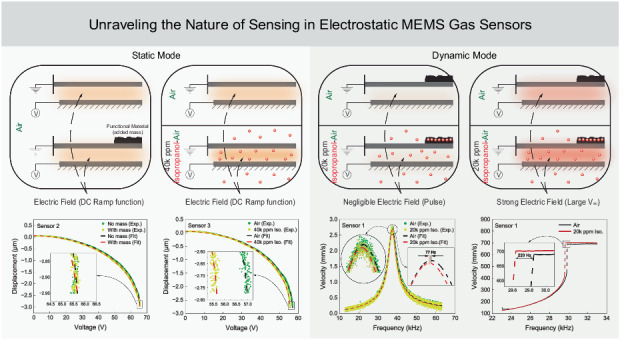

## Introduction

Extensive research spanning the past two decades has revealed the superiority of Micro Electromechanical Systems (MEMS) sensors over conventional counterparts. This is attributed to their small size, lightweight, high accuracy, portability, affordability, and their real-time operational capability^1^. Consequently, they found widespread adoption in a multitude of applications, such as pressure sensors^2^, temperature sensors^3^, force sensors^4^, accelerometers^5^, gyroscopes^6^, and gas sensors^7–15^.

Electrostatic transduction stands out as a popular choice in MEMS sensors due to its speed, low power consumption, independence from external field sources^16^, and ease of fabrication and integration with CMOS circuits^17^. These sensors are functionalized by coating them with detector materials, such as polymers^7,8,15^, metal oxides^14^, or metal organic frameworks (MOFs)^9^. These materials capture and sorb particles of the target analyte from the surrounding environment and result in a detection signal, a measurable change of the sensor response.

These sensors have traditionally been described as mass^7,15,18^, gravimetric, or inertial^8,9,19,20^ sensors. They typically utilize one of two modes of detection: static or dynamic. In static detection, the sensor measures a change in static deflection in the presence of the target gas^7,15,21^. In dynamic detection, the sensor measures a shift in resonant frequency or location of a bifurcation^8,11,22^ in the presence of the target gas.

Until now, those detection modes have been perceived in the literature as mass dependent and the sensors as, in effect, gravimetric sensors due to their reliance on the mass of sorbed gas. In this paper, we systematically investigate this proposition by designing and conducting a series of experiments on two classes of electrostatic MEMS sensors to examine their response to changes in mass and isopropanol vapor exposure, in both the static and dynamic detection modes. Our findings promise to improve our understanding of the sensing mechanisms underlying electrostatic MEMS inertial gas sensors.

## Results and discussion

### Sensors design and fabrication

**Fig. 1:**
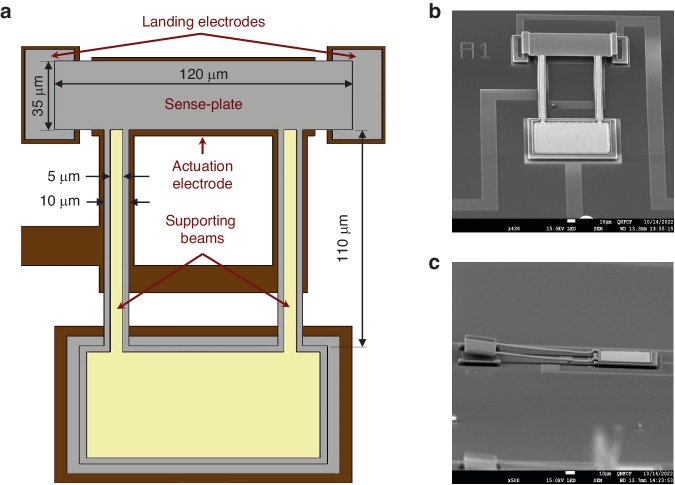
Sensor design. **a** A shematic diagram of the PolyMUMPs sensors. **b**, **c** Scanning Electron Microscope (SEM) top and side views of the sensor, respectively

This study utilizes two classes of MEMS sensors fabricated using the PolyMUMPs process^23^ and an in-house silicon-on-insulator (SOI) process^24^. The PolyMUMPs sensors^25^, Fig. 1, are comprised of a sense-plate (35 *μ*m × 120 *μ*m) plate, supported by two beams (110 *μ*m × 10 *μ*m) fabricated in the 1.5 *μ*m thick Poly 2 structural layer. An actuation electrode is patterned onto the substrate in the Poly 0 layer under the center 90 *μ*m of the plate and support beams. Two identical landing electrodes are also patterned on either side of the actuation electrode in the Poly 1 structural layer. The height differential between the actuation electrode and landing electrodes serves to prevent contact between the plate and the actuation electrode, thereby reducing the possibilities for stiction, dielectric charging, and short circuits.

To reduce energy dissipation due to squeeze-film damping, a 5 *μ*m wide and 500 nm thick gold layer was deposited along the two support beams of the PolyMUMPs sensor. The gold layer induces an upward curvature in the beams due to differences in the coefficients of thermal expansion between gold and polysilicon. The resulting upward curvature of the beams can be seen in the side view shown in Fig. 1c. This technique increases the capacitive gap, and the resonator’s quality factor.

The SOI sensors, Fig. S1, are designed to move in-plane^24^. The sensors were fabricated in an SOI wafer with a 30 *μ*m thick crystal silicon device layer and a 2 *μ*m buried oxide layer. Each sensor consists of a sense-plate (80 *μ*m × 10 *μ*m) supported by two beams (370 *μ*m × 3 *μ*m). The capacitive distance between a side electrode and the plate and beam sides is 3 *μ*m.

### Characterization protocol

Each PolyMUMPs sensor in this study underwent an experimental protocol to measure its characteristics. This section describes the protocol and its results for sensor #1. Similar results were also obtained for sensors #2 and 3.

**Fig. 2:**
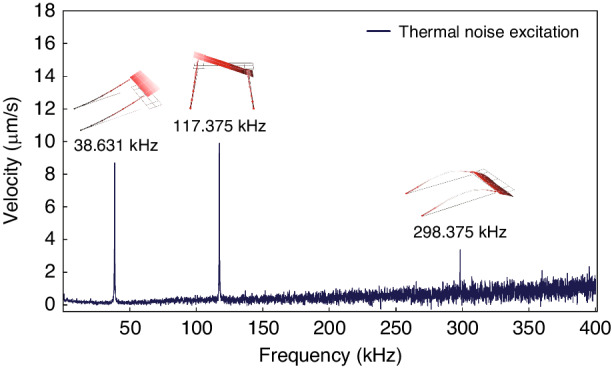
Modal response. The Fast Fourier Transform (FFT) of the sense-plate tip’s right corner velocity under thermo-mechanical noise excitation and the mode shapes corresponding to the peaks appearing in the frequency spectrum

As described in section ‘Modal Response’, the modal response of the sensor was obtained under thermo-mechanical noise excitation by placing it within a vacuum chamber. A Laser Doppler Vibrometer (LDV) was used to measure the out-of-plane velocity of the sense-plate tip’s right corner. The frequency response of the sensor, Fig. 2, was evaluated by taking the Fast Fourier Transform (FFT) of the time-domain measurements. In the frequency range up to 400 kHz, three distinct peaks are observed, locating the first three natural frequencies. The mode shapes corresponding to those frequencies were identified experimentally, using a multi-point LDV scan, as the first out-of-plane bending mode at *f*_1_ = 38.631 kHz, the first torsional mode at *f*_2_ = 117.375 kHz, and the second out-of-plane bending mode at *f*_3_ = 298.375 kHz. The mode shapes are shown above their peaks in Fig. 2.

**Fig. 3:**
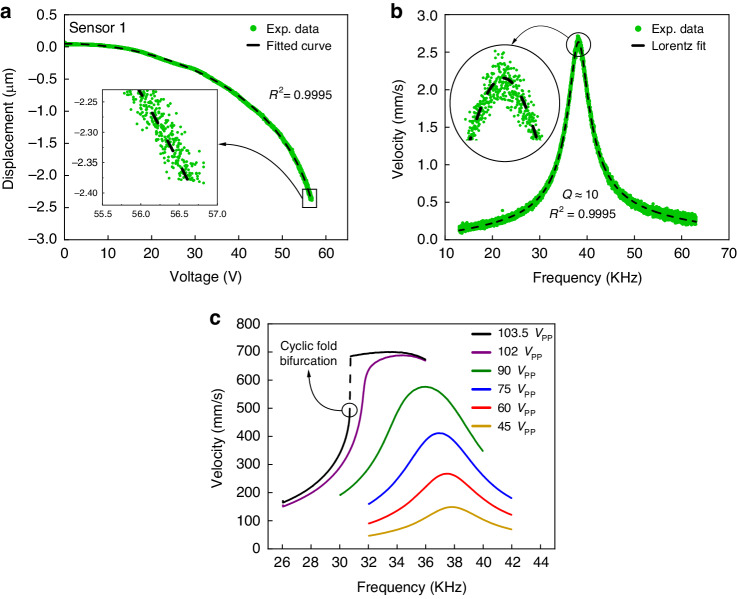
Characterization of sensor #1. **a** The measured and fitted voltage-displacement relationship at the sense-plate center. **b** The frequency response of the sensor at the sense-plate center under a pulse train. **c** The frequency-response curves of the sensor at six levels of harmonic excitation

The static and dynamic baseline responses of the sensor were obtained within the test chamber under standard test conditions, namely a dry air flow rate of 100 sccm, room temperature, a pressure of 1.02 bar, and a relative humidity of 10–15%. As described in section ‘Static Detection’, a quasi-static actuation scheme was employed to evaluate the static relationship between the dc voltage *V* and the displacement *d* of the sense-plate center. The voltage results in an attractive electrostatic force between the plate and the actuation electrode, as described by^26,27^:

1 $${F}_{e}\propto \frac{\varepsilon A}{{(g-d)}^{2}}{V}^{2}$$

where *ε* is the permittivity of air, *A* is the active sense-plate area, *g* is the unactuated gap between the sense-plate and the actuation electrode, and *d* is the displacement of the sense-plate. Figure 3a shows the measured (green dots) and a functional fit (dashed line) of the voltage-displacement relationship of the sense-plate center.

Dynamic characterization was focused on the fundamental mode targeted for the implementation of a bifurcation sensor. A pulse train was applied to the sensor, and the velocity of the sense-plate center was measured using the LDV, as per section ‘Dynamic Detection’. A Lorentzian fit (dashed line in Fig. 3b) was created out of the FFT of the measured velocity (green dots) to estimate the resonant frequency and the quality factor of the sensor in air as *f*_∘_ = 38.188 kHz and *Q* ≈ 10, respectively.

The frequency response of the sensor in the vicinity of the resonant frequency was obtained under an unbiased harmonic signal via a forward sweep of the excitation frequency, as explained in section ‘Dynamic Detection’. Figure 3c shows the frequency response of the sensor for six levels of the peak-to-peak voltage (*V*_pp_) varying from 45 to 103.5 V. As the electric field intensifies, the resonant peak of the frequency-response curve bends to the left, exhibiting a softening nonlinearity. Beyond *V*_pp_ = 102 V, multivaluedness appears in the sensor response. Under a voltage waveform with *V*_pp_ = 103.5 V, a jump occurs at *f* = 30.7 kHz, where cyclic fold bifurcation sends the response from a lower (small) branch to an upper (resonant) branch.

### Static detection mode

**Fig. 4:**
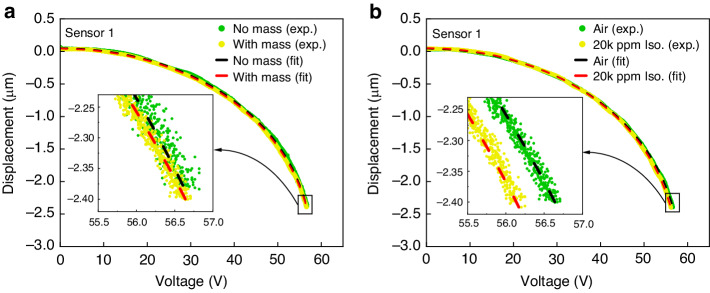
Static Detection, experiment #1. The experimental (dots) and fitted (dashed lines) voltage-displacement curves. **a** Sensor #1 in air: bare versus functionalized with PANI and **b** Functionalized sensor #1: in air versus exposed to 20,000 ppm isopropanol vapor

In this section, we compare the response of the PolyMUMPs sensor to deposition of a solid mass versus exposure to isopropanol vapor. An added mass *δm* of Polyaniline (PANI) doped with 5% ZnO was deposited on the sense-plate of sensor #1, as described in section ‘Sensor Functionalization’. As a result, the natural frequency dropped by *δf* = 800 Hz. Assuming linear vibrations, we used the relationship:

2 $$N=\frac{\delta m}{{m}_{s}}={\left(\frac{{f}_{1}}{{f}_{1}-\delta f}\right)}^{2}-1$$

where *m*_*s*_ is the effective mass of the sensor, to estimate the fractional mass of the deposited PANI as *N* = 4.25%. Using the procedure described in section ‘Static Detection’, we compare voltage-displacement relationship for sensor #1 before and after deposition of PANI in Fig. 4a. The results do not reveal a measurable difference between the two sets of response even for higher voltages close to pull-in where the sensor is more susceptible to small perturbations. The curve fits of the voltage-displacement relationship show a maximum difference of 30 mV in the voltage required to realize a displacement of 2.4 *μ*m, which lies within measurement error.

Under the same test conditions, the sensor was exposed to a mixture of 20,000 particles per million (ppm) isopropanol vapor and air. Following the procedure of section ‘Static Detection’, we obtained the sensor’s voltage-displacement relationship. We compare in Fig. 4b the voltage-displacement relationships of the isopropanol sensor in baseline air and in a concentration of 20,000 ppm isopropanol vapor. The results demonstrate a drop of 0.5 V in voltage required to realize a displacement of 2.4 *μ*m. This voltage difference is one order-of-magnitude larger than the measurement error.

Similar results were obtained for sensor #2. In this case, deposition of PANI resulted in a frequency drop of *δf* = 2100 Hz equivalent to a fractional mass of *N* = 11%. The difference between the voltage required to realize a displacement of 2.95 *μ*m before and after deposition of this mass was within measurement error, Fig. S2a. On the other hand, the difference between the voltage required for the sensor to realize the same displacement in baseline air and in a concentration of 30,000 ppm isopropanol vapor was 0.9 V, Fig. S2b.

We deployed sensor #3 in a another experiment to test the change-in-mass hypothesis. In this case, we compare the pull-in voltage of the sensor in a flat position, where the full weight of the sense-plate results in an equilibrium position closer to the substrate, and inclined at 60^∘^ with respect to the flat position, thereby reducing the out-of-plane weight component by 50%.

**Fig. 5:**
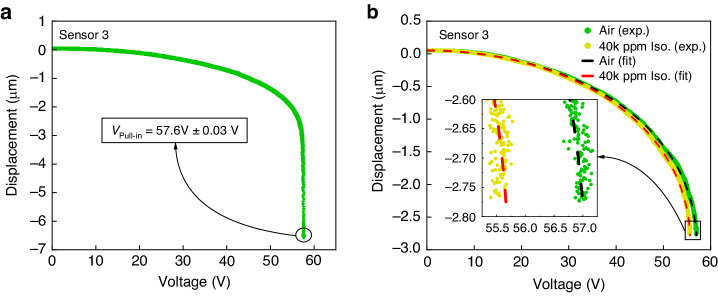
Static detection, experiments #2 & #3. **a** The measured voltage-displacement relationship for sensor #3 up to pull-in. **b** The measured and fitted voltage-displacement relationship for sensor #3 before and after exposure to 40,000 ppm isopropanol vapor

Figure 5a presents the measured voltage-displacement relationship up to static pull-in, where the sense-plate jumps to touch the landing electrodes, at the flat position. The pull-in voltage was measured as 57.6 ± 0.03 V for both the flat and inclined configurations. This shows that the change in the sensor equilibrium position caused by a 50% reduction in the active sensor weight was insufficient to induce a measurable changes in the capacitive gap and, thus, pull-in voltage.

In a third experiment, we compared the response of the bare sensor #3 to air versus a concentration of 40,000 ppm isopropanol vapor. Figure 5b shows the measured (dots) voltage-displacement relationship and its functional fit (dashed lines) before and after exposure to isopropanol vapor. A leftward shift in the voltage-displacement curve towards lower voltages is observed when exposed to isopropanol, resulting in a drop of 1.3 V in the voltage required to realize a displacement of 2.75 *μ*m.

A fourth experiment was conducted using two SOI sensors. The experiment involved measuring the static pull-in voltage of each sensor in vertical and horizontal orientations. In the former case, the full weight of the sensor acts in the same direction as the electrostatic force, towards the actuation electrode. In the latter case, the weight of the sensor is completely eliminated from the force balance, Fig. S1.

Table S1 lists the measured static pull-in voltage in each case. The results demonstrate that the static pull-in voltage was unchanged, irrespective of the sensor orientation. This means that the presence or absence of the sensor full weight did not change the capactive gap to the extent of creating a measurable difference in the static pull-in voltage within the measurement level of precision ( ± 30 mV).

We conclude that the added mass of deposited PANI and the reduction or elimination of the weight component acting on the sensor do not have a measurable effect on the static deflection of the plate, the capacitive gap, the electrostatic force, or the voltage-displacement relationship. Considering that the added and reduced masses in question are at least one order-of-magnitude larger than the mass added by sorption of isopropanol, this precludes the proposition that static detection of isopropanol by electrostatic MEMS sensors is related to changes in mass. On the other hand, the sensors’ voltage-displacement relationship underwent a measurable change in the presence of isopropanol vapor even in the absence of a functional material. Examining the electrostatic force expression, Eq. (1), suggests that the vapor is increasing the permittivity of the medium, leading to an increase in the electrostatic force. Therefore, the underlying capacitive sensing mechanism in this case is indeed permittivity-based.

### Dynamic detection mode

Two experiments were conducted to investigate the response of the PolyMUMPs sensors to isopropanol vapor in the absence and presence of electrostatic fields. Experiment #5 embodies a frequency shift sensor where variations in the resonant frequency of the fundamental mode are observed to detect the concentration of isopropanol vapor. In this case, a pulse train with a negligible electrostatic field was adopted to excite the sensor. Experiment #6 embodies a bifurcation sensor where a large voltage signal, a strong electrostatic field, is used to excite large motions and multivalued responses. Shifts in the frequency of a bifurcation are then used to measure the concentration of isopropanol vapor.

**Fig. 6:**
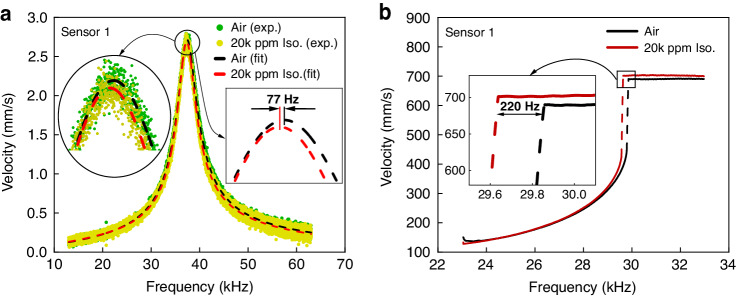
Dynamic detection, experiments #5 & #6. **a** The measured and fitted impulse-response and **b** the frequency-response curves under an unbaised voltage waveform with *V*_pp_ = 103.5 V for sensor #1 in air and in air mixed with 20,000 ppm isopropanol vapor

Experiment #5 was conducted following the procedure described in section ‘Dynamic Detection’ to obtain the impulse-response of sensor #1 in air and in air mixed with 20,000 ppm of isopropanol vapor, Fig. 6a. We found that the resonant frequency of the fundamental mode dropped by *δf* = 77 Hz due to the added mass of sorbed isopropanol vapor. This corresponds to a fractional mass of *N* = 0.4%. Similarly, we found that the resonant frequency of sensor #2 decreased by *δf* = 192 Hz after exposure to 40,000 ppm of isopropanol vapor, Fig. S3a, corresponding to a fraction mass of *N* = 1%.

Experiment #6 followed the procedure described in section ‘Dynamic Detection’ to measure the shift in the location of a cyclic-fold bifurcation in the vicinity of primary resonance of sensor #1 as the medium was changed from air to air mixed with 20,000 ppm of isopropanol vapor, Fig. 6b. The bifurcation frequency of sensor #1 dropped by *δf* = 220 Hz upon exposure to isopropanol vapor, about three times the drop in experiment #5. Similarly, we found that the cyclic-fold bifurcation frequency of sensor #2 decreased by *δf* = 710 Hz after exposure to 40,000 ppm of isopropanol vapor, Fig. S3b, which is also threefold that obtained in experiment #5.

Najar et al.^28^ found that the shifts in the natural frequency and bifurcation location of inertial sensors due to added masses are similar in size. We, therefore, postulate that the threefold difference we observed here is due to the absence of electrostatic fields in experiment #5 and their presence in experiment #6. In the former case, the sensor responds only to added mass. In the latter case, the sensor responds to both added mass and perturbations in the electrostatic field. A strong electrostatic field results in a larger downward shift in the bifurcation location due to strong softening nonlinearities.

To determine whether the perturbation in the field are due to increased permittivity or changes in the charge distribution across the field, we measured the bias voltage in experiment #6 before and during exposure to the gas mixture and found it unchanged. This suggests that the field perturbations are due to an increase in the permittivity of the medium in the presence of isopropanol. Therefore, we can assert that the sense mechanism of the dynamic mode in electrostatic MEMS sensors is a combination of mass and perturbations of the electrostatic field.

## Conclusions

We carried out a comprehensive investigation to understand the sensing mechanism in electrostatic MEMS inertial gas sensors using two classes of sensors fabricated by a commercial foundry, PolyMUMPs, and an in-house SOI process. First we investigated the static detection mode and found that the weight of the deposited functional material and the sensor itself had no measurable effect on the sensor displacement. On the other hand, exposure to isopropanol vapor even in the absence of a detector material results in a change in the sensor voltage-displacement relationship. These findings challenge the classification of these sensors as ‘gravimetric’ sensors and provide compelling evidence that their capacitive sensing mechanism is permittivity-based.

We also investigated the sensing mechanism in both types of the dynamic detection mode, namely shifts in a resonant frequency and the location of a bifurcation. Our results show that the presence of an electrostatic field triples the measured frequency shift in the presence of isopropanol vapor. This enhancement was attributed to the vapor’s ability to increase the medium permittivity, thereby presenting compelling evidence that this sense mechanism combines response to changes in mass and permittivity of the medium. The predominance of either sorbed mass or permittivity change depends on the properties of the functional material and the sensor design.

It is expected that the two sensing mechanisms will have different response and recovery time constants. Understanding those dynamics will be crucial for optimizing sensor performance. Specifically, the adsorption time constant depends on the characteristics of the functional material and its responsivity and affinity to the target gas. In contrast, permittivity changes manifest immediately upon the introduction of vapor into the capacitive gap, suggesting different time constants for the two mechanisms.

This work contributes to a deeper understanding of the behavior of these sensors and highlights the potential for better gas sensor designs. This is particularity the case for dynamic mode sensors where the sensor design must take into account the responsivity to both mass and permittivity.

Specifically, our results show that improving responsivity of those sensors can be achieved by changing the functional layer thickness and coverage area^29^ to increase sorbed mass or reducing the size of the capacitive gap to enhance sensitivity to permittivity changes. Our findings also indicate that a possible route to improve the design of inertial electrostatic gas sensors is to introduce the functional layer into the capacitive gap. In this case, swelling of the functional layer due to analyte sorption will increase the medium permittivity by reducing the air gap^30^. In addition, mixing of the analyte with air will increase the mixture’s permittivity. The combination of the two effects will, therefore, amplify permittivity change as a sensing mechanism. Finally, our findings show that inertial electrostatic sensing is in fact an extension of traditional capacitive sensing. While capacitive sensing measures explicitly the change in the electrostatic field^30^, inertial electrostatic sensing measures its acoustic effects, namely changes in the sensor stiffness.

## Materials and methods

### Experimental setup

**Fig. 7:**
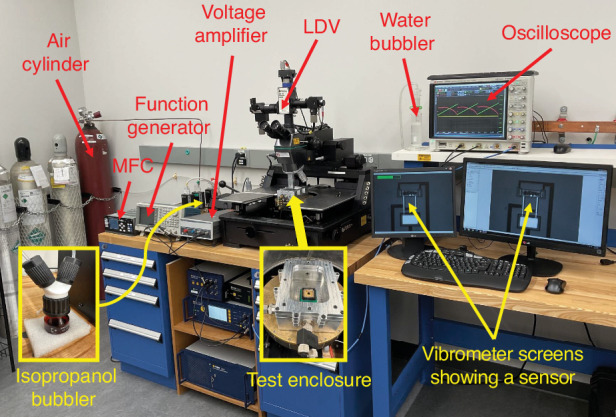
Experimental setup. A view of the experimental setup used for sensor characterization and testing, showing the test chamber, air cylinder, isopropanol and water bubblers, mass flow control system, signal generators, the LDV, and the discretization oscilloscope

The experimental setup utilized in characterization and testing of the gas sensors is shown in Fig. 7. A custom-designed aluminum chamber serves as a test enclosure. An anti-reflective glass port provides optical access. It features two ports for gas flow as well as an electrical port to deliver the actuation signal. An environmental sensor, SensorPush^31^, is placed inside the chamber to continuously monitor environmental conditions throughout the experiment.

The drive signal is generated by a combination of a function generator, Tektronix AFG3022B^32^, and a 15x voltage amplifier. The gas inlet is controlled via a mass flow control system (MFC), MKS Series 946^33^, enabling precise control over the flow of air and mixing with isopropanol vapor. To prevent potential backflow, the outlet port is connected to a water bubbler.

The test enclosure, including the sensor chip, is placed under the turret of an LDV, Polytec (MSV 400)^34^, to measure the sensor response. The output voltage of the photodetector is discretized using a Keysight oscilloscope (DSOS204A)^35^.

### Modal response

With the sensor chip placed inside the test chamber, the inlet port was connected to a vacuum pump to bring the pressure down to 6 mbar. The sensor’s higher quality factor, realized at low pressure, elevates the resonant responses to thermal noise above the (measurement) noise floor. The LDV was employed to measure the velocity at the tip of the sense-plate right edge, capturing motions associated with out-of-plane bending and torsional modes. The time-domain response was transformed into the frequency domain using an embedded FFT analyzer within the LDV software interface. Peaks within the initial 400 kHz of the spectrum were examined to identify the first three natural frequencies.

To identify the vibration modes associated with the first three natural frequencies, sensor #1 was subjected to a harmonic (electrostatic) excitation force at a frequency equal to the natural frequency under investigation. A multi-point scan using the LDV was carried out to capture the motions over a grid covering the surface of the sensor. Using the vibrometer interface software, the mode shapes were evaluated and visualized as shown in Fig. 2.

### Static detection

Dry air was introduced into the chamber at a flow rate of 100 sccm until stable environmental conditions - temperature, humidity, and pressure - were consistently achieved. A saw-tooth voltage signal, with a frequency of 200 Hz ≪ *f*_∘_, was applied to the actuation electrode, while the sensor and landing electrodes were grounded. The resulting displacement of the sense-plate center was measured using the LDV. The measured response and the voltage signal were digitized using the oscilloscope. The driving signal amplitude was gradually increased to achive large displacements close to but less than the pull-in instability. The digitized data were processed using MATLAB to obtain a fit relating displacement to voltage following the functional form:

3 $$V=\frac{3{V}_{{{{\rm{p}}}}}}{g}\,\sqrt{\frac{3}{4g}({n}_{d}{(g-{n}_{d})}^{2}+(g-3{n}_{d})(g-{n}_{d})d-(2g-3{n}_{d}){d}^{2}+{d}^{3})}$$

where *V* is the voltage signal, *V*_p_ is the pull-in voltage, *g* is the unactuated gap between the plate and the actuation electrode, *d* is the measured displacement, and *n*_*d*_ is displacement noise. The coefficient of determination of the fit was *R*^2^ = 0.9995, indicating high fitting accuracy.

### Dynamic detection

To demonstrate a frequency shift sensor, we employed a pulse train to obtain its impulse response. The velocity of the sense-plate center was measured using the LDV. Subsequently, the frequency-response curve was obtained by taking the FFT of the measured time-domain response using MATLAB. A Lorentzian fit was then applied to determine the sensor’s resonant frequency and quality factor using the following form:

4 $$v=\frac{A}{Q\sqrt{{(1-{(\frac{f}{{f}_{c}})}^{2})}^{2}+{(\frac{f}{{f}_{c}Q})}^{2}}}+{n}_{f}f+n$$

where *v* is the measured velocity, *A* is the amplitude of the Lorentzian curve, *Q* is the quality factor, *f*_*c*_ is the center or resonant frequency, f is the variable frequency at which the Lorentzian function is evaluated, *n*_*f*_ is frequency-dependent noise, and *n* is white noise. The coefficient of determination of the fit was *R*^2^ = 0.9995, showing an accurate fit.

For sensor #1, we set the pulse parameters as follows: a pulse period of 1.024 seconds, a low duty cycle of 0.001%, an amplitude of 30 volts, and an LDV sampling frequency of 0.512 MHz. For sensor #2, we employed the following pulse settings: a pulse period of 16 milliseconds, a low duty cycle of 0.005%, an amplitude of 22.5 volts, and an LDV sampling frequency of 1.024 MHz. The sampling rate was selected at more than six times the Nyquist rate of the fundamental mode to obtain a good quality for the measured signal.

To obtain the forced frequency response in the vicinity of the primary resonance and to demonstrate a bifurcation sensor, we adopted electrostatic excitation by applying an unbiased voltage waveform described by the equation:

5 $$V(t)={V}_{{{{\rm{ac}}}}}\cos (2\pi {f}_{{{{\rm{ac}}}}}t)$$

Here, *f*_ac_ represents the signal frequency, and *V*_ac_ denotes the amplitude of the voltage signal. The resulting electrostatic force *F*_*e*_(*t*), expressed as:

6 $${F}_{e}(t)=\beta {V}_{{{{\rm{ac}}}}}^{2}\left(1+\cos (2\pi (2{f}_{{{{\rm{ac}}}}})t)\right)$$

where *β* is a function of the capacitive gap, the permittivity of the medium, and the effective capacitance area, exhibits harmonic behavior with a frequency twice that of the signal frequency. Therefore, the signal frequency *f*_ac_ was swept, in a forward sweep, in the vicinity of ($\frac{1}{2}{f}_{\circ }$) at a rate of 2.5 kHz/sec. Subsequently, we measured the velocity of the sense-plate center using the LDV and digitized it using the oscilloscope. To induce a multivalued response leading to bifurcation, we set the signal peak-to-peak voltage (*V*_*p*−*p*_ = 2*V*_ac_) to 103.5*V* for sensor #1 and 100.95*V* for sensor #2. Finally, we obtained the frequency-response curves by evaluating the RMS of time-domain response using Mathematica, knowing the start and end frequencies.

### Sensor functionalization

PANI and PANI doped with ZnO have affinity towards various volatile substances, such as methanol, isopropanol, and benzyl alcohol^36–38^. Isopropanol was chosen as the test vapor due to its widespread use in pharmaceuticals, cosmetics, and cleaning products, making it readily available and good representative of volatile organic gases.

**Fig. 8:**
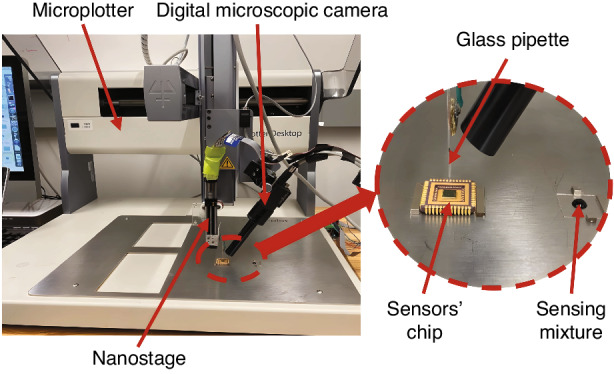
Deposition system. A view of the system used for the deposition of PANI doped with 5% ZnO, showing the microplotter for coarse micromanipulation, the nano stage for precise vertical positioning of the attached glass pipette, and a digital microscopic camera for real-time process monitoring

PANI doped with 5% ZnO, in the form of solid powder, was stirred in a carrier fluid, ethylene glycol, at a concentration of 10–15% by weight. The mixture acts as a carrier of the powder to its target location. We employed a semi-automated deposition system^39,40^ to deposit it on the sense-plate (Fig. 8). Ethylene glycol was then allowed to naturally evaporate, leaving behind a residue of PANI on the sense-plate. This process was repeated until 30–80% of the sense-plate surface was coated in accordance with the experimental protocol.

### Supplementary information

Supporting file
