# Supplementary material for: Unraveling the nature of sensing in electrostatic MEMS gas sensors
Source: Microsyst Nanoeng. 2024 May 6;10:56. doi: 10.1038/s41378-024-00688-3 (PMC11074296; doi:10.1038/s41378-024-00688-3)
Supplement: Supplementary file 1 — Supporting file [file 41378_2024_688_MOESM1_ESM.pdf]

Supplementary Information for
Unraveling the Nature of Sensing in Electrostatic MEMS Gas Sensors

Yasser S. Shama[1,2*], Sasan Rahmanian[1], Hamza Mouharrar[1], Rana Abdelrahman[1], Alaaeldin Elhady[1], Eihab M. Abdel-Rahman[1]

[1*]Systems Design Engineering, University of Waterloo, 200 University Ave W, Waterloo, N2L 3G1, ON, Canada.
[2]Mechanical Engineering, Benha Faculty of Engineering, Benha University, Benha, 13511, Egypt.

*Corresponding author(s). E-mail(s): Ys2shama@uwaterloo.ca;

1 Sensors Design and Fabrication

The scanning electron microscope (SEM) image of the SOI sensor used in experiment # 4 is shown in Fig. S1.

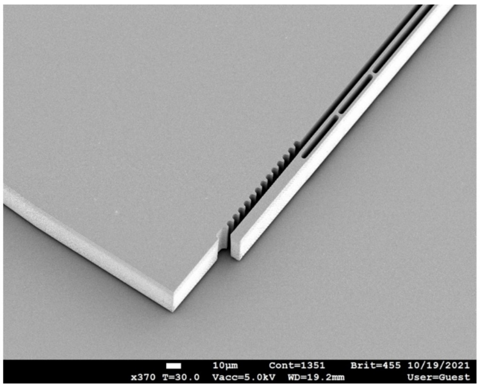

Fig. S1: SEM image for the SOI sensor

2 Static Detection Mode

Fig. S2(a) shows that, for polyMUMPs sensor # 2, the difference between the voltage required to realize a displacement of 2.95 µm before and after deposition of PANI with a fractional mass of $N = 11\%$ was within measurement error. However, Fig. S2(b) shows that upon exposure to air mixed with 30,000 ppm isopropanol vapor, there was a notable drop of 0.9 V in the voltage required by the sensor to realize the same displacement, compared to the baseline air condition.

The results of experiment # 4 is tabulated in Table S1. It lists the Pull-in voltages for two SOI Sensors in vertical and horizontal orientations.

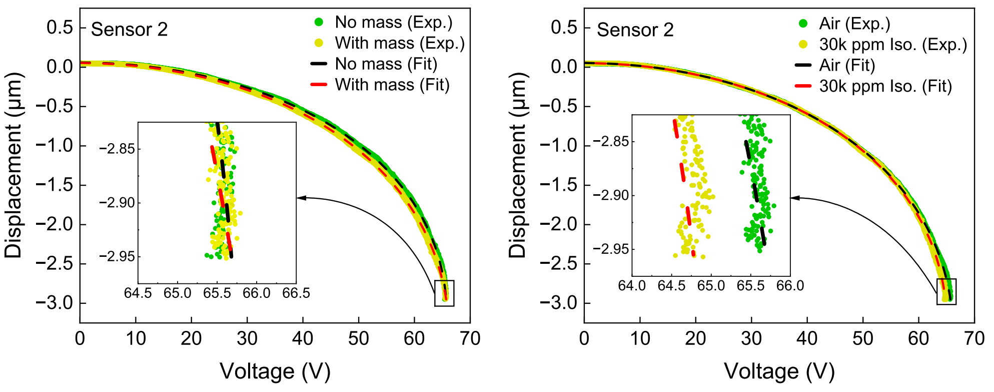

(a) (b)

Fig. S2: Static Detection, experiment # 1. The experimental (dots) and fitted (dashed lines) voltage-displacement curves of (a) sensor # 2 in air: bare versus functionalized with PANI, (b) functionalized sensor # 2: in air versus exposed to 30,000 ppm isopropanol vapor.

Table S1: SOI sensors, experiment # 4. Measured static pull-in voltage in the vertical and horizontal orientations

Sensor	Trial	Vertical	Horizontal
1	1	28.935V	28.935V
	2	28.935V	28.935V
2	1	31.410V	31.410V
	2	31.410V	31.410V
	3	31.410V	31.410V

3 Dynamic Detection Mode

Fig. S3(a) shows the impulse response of polyMUMPs sensor # 2 in air and in air mixed with 40,000 ppm of isopropanol vapor, which displays a drop of $\delta f = 192\,\text{Hz}$ in the resonant frequency after exposure to the analyte, corresponding to a fractional mass of $N = 1\%$. Fig. S3(b) shows that the cyclic-fold bifurcation frequency has dropped by $\delta f = 710\,\text{Hz}$ after exposure to 40,000 ppm of isopropanol vapor which is threefold the shift obtained in Fig. S3(a).

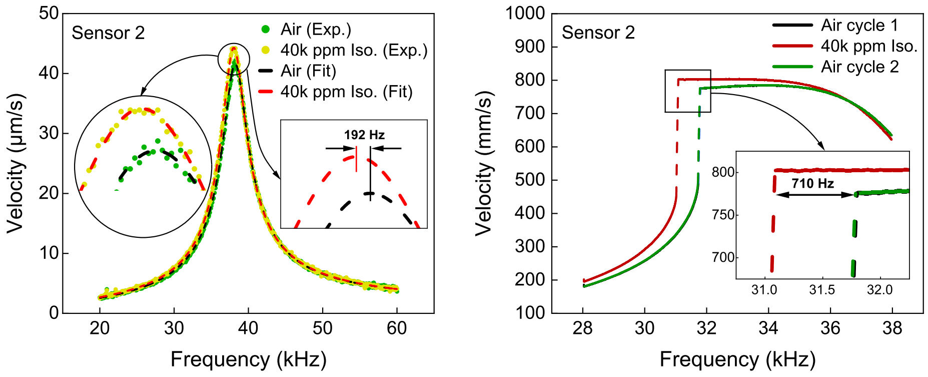

(a) (b)

Fig. S3: Dynamic Detection, experiments # 5 & # 6. (a) The measured (dots) and fitted (dashed lines) impulse-response and (b) the frequency-response curves under an unbaised voltage waveform with $V_{\text{pp}} = 100.95\,\text{V}$ for sensor # 2 in air and in air mixed with 40,000 ppm isopropanol vapor. The black and green frequency-response curves in (b) show sensor reversibility.
